# A study on the electrocardiography in dogs: Reference values and their comparison among breeds, sex, and age groups

**DOI:** 10.14202/vetworld.2020.2216-2220

**Published:** 2020-10-23

**Authors:** Joydip Mukherjee, Smruti Smita Mohapatra, Sonali Jana, Pradip Kumar Das, Prabal Ranjan Ghosh, Kinsuk Das and Dipak Banerjee

**Affiliations:** Department of Veterinary Physiology, Faculty of Veterinary and Animal Sciences, West Bengal University of Animal and Fishery Sciences, Kolkata, West Bengal, India

**Keywords:** dogs, electrocardiogram, heart rate, rhythm

## Abstract

**Aim:**

The present investigation was intended to generate some basic data on electrocardiography (ECG) parameters in different breeds and their alterations in respect to sex and age.

**Materials and Methods:**

The present investigation was carried out on 239 owned dogs of 11 different breeds presented to the Institute Veterinary Clinic during 2018-2019. The animals, irrespective of breed and sex were grouped on the basis of their age groups. Recordings of rdiartR, BPL, India) at 25 mm/s paper speed and 10 mm=1 mV calibration keeping the animals on the right lateECG were performed by a single-channel ECG machine (Caral recumbence without any anesthesia. Heart rate, along with the amplitude and duration of different waves and complexes was measured.

**Results:**

Heart rate did not vary significantly among breeds, sex, and different age groups. The highest heart rate has been reported in Doberman and the lowest in Beagle. The heart rate was lowest at the age group of 6 months-2.5 years and highest around 10.5-12.5 years irrespective of breed and sex. The incidence of sinus arrhythmia was mostly seen in older dogs. All the waves and complexes did not vary significantly between breeds, age, and sex except P duration which was significantly (p<0.05) higher in Golden retriever and Doberman breeds.

**Conclusion:**

The present investigation generated some reference values of ECG in dogs which will help the clinicians to diagnose different cardiac abnormalities through ECG.

## Introduction

Cardiac diseases, a silent killer, are common in dogs. The reports of American Veterinary Medical Association stated that 1 in 10 dogs suffer from cardiac diseases [1]. In India, the prevalence rate of cardiac diseases in canines is also increasing at in fast rate [2]. Routine monitoring of cardiac functions is, therefore, an essential prerequisite for early diagnosis of cardiac abnormalities. Electrocardiography (ECG), a non-invasive and inexpensive techniques, is widely employed for the determination of heart rate, heart rhythm, conduction integrity, and mean electrical axis together with myocardial and pericardial affections along with monitoring certain non-cardiac abnormalities such as electrolyte imbalance, drug toxicity, and hormonal disturbances [3,4].

Scanty literatures are available on the ECG in pets in India compared to other developed countries [5,6]. Incidence and risk assessment of cardiac arrhythmias in 374 dogs of 15 different breeds with respect to age, breed, and sex and associated biochemical changes were carried out by Kumar *et al*. [7]. Gugjoo *et al*. [8] reported reference values of six limb lead ECG in 24 healthy conscious Labrador Retriever dogs. We, while, investigating the ECG pattern of three exotic breeds of trained dogs [9], encountered difficulties due to lack of appropriate reference values based on a large number of dog populations in respect to breed, sex, age, and other physiological states for accurate interpretation and appropriate therapeutic interventions [10].

Therefore, the present investigation was intended to generate some basic data on ECG parameters based on 239 dogs of 11 different breeds in respect to sex and age. Breed- and age-wise incidence of different cardiac abnormalities was also discussed.

## Materials and Methods

### Ethical approval

The experiments on animals including all procedures of this study were approved by Institutional Animal Ethics Committee (Registration number: 763/03/a/CPCSEA).

### Study location, period and clinical subjects

The present investigation was carried out on the 239 owned dog patients presented to the Veterinary Clinic under West Bengal University of Animal and Fishery Sciences, Kolkata, West Bengal, India, during 2018-2019 by the clients. There were 11 different breeds, namely, Mongrel (n=21), Labrador Retriever (n=26), crossbred (n=25), Spitz (n=22), Beagle (n=17), Pug (n=29), Dachshund (n=15), German Shepherd dog (n=24), Pomeranian (n=26), Golden Retriever (n=20), and Doberman (n=14). The average age was 6.9 years. Out of the total studied dogs, 42.86% (n=103) were male and 57.14% (n=136) were female. The animals irrespective of breed and sex were grouped on the basis of their age into below 6 months (n=18), 6 months-2.5 years (n=35), 2.5 years-4.5 years (n=34), 4.5 years-6.5 years (n=33), 6.5 years-8.5 years ((n=36), 8.5 years-10.5 years (n=37), 10.5 years-12.5 years (n=25), and 12.5 years-15 years (n=21).

### Recording of ECG

Recordings of ECG were performed by a single-channel ECG machine (CardiartR, BPL, India) at 25 mm/s paper speed and 10 mm=1 mV calibration keeping the animals on the right lateral recumbence without any anesthesia. All the recordings of standard bipolar limb leads (I, II, and III) and unipolar augmented limb leads (aVR, aVL, and aVF) were taken on thermosensitive ECG paper (CardiartR) having width of 50 cm and recording width of 40 mm. Each small square on horizontal axis represents 0.04 second and on vertical axis represents 0.1 millivolt (mV). Amplitude of P, Q, R, S, and T waves was measured together with PR interval, QRS interval in lead II as its electrode direction matched the typical waves as the depolarization vector [11].

### Measurement of heart rate, complexes, and intervals of ECG

Waves and complexes of lead-II were used as typical waves to calculate amplitude and durations of different waves and complexes as the depolarization vector is directed toward the electrode of lead-II [11]. Amplitude and duration of P, Q, R, S, and T waves were measured together with PR interval, QRS interval, and QT interval. Heart rate was calculated by successive R-R interval.

### Statistical analysis

Three-way analysis of variance was used to test the significance among breeds, age groups, and sex by SPSS software version 15.0 (SPSS, Inc., Chicago, IL, USA).

## Results and discussion

### Heart rate

The heart rate (bpm) in different breeds of dogs is presented in Table-1. No significant differences between different breeds of dogs in respect to heart rate were found though the highest heart rate (150.00±18.24) has been observed in Doberman and the lowest in Beagle (100.00±7.34). Sex has no significant effect on the heart rate, irrespective of age and breeds. The heart rate was lowest at the age group of 6 months-2.5 years and highest around 10.5-12.5 years irrespective of breed and sex.

**Table 1:** The mean with standard error of various electrocardiography parameters of dog.

Group	HR (bpm)	P_amp_ (mv)	P_dur_ (s)	PR interval (s)	QRS_dur_ (s)	R_amp_ (mv)	QT interval (s)	T_dur_ (s)	T_amp_ (mv)
Overall	119.24±4.4	0.25±0.01	0.04±0.01	0.07±0.01	0.04±0.01	1.31±0.07	0.19±0.01	0.04±0.01	0.22±0.014
Variation between breeds									
Mongrel	108±18.5	0.25±0.03	0.04^b^±0.01	0.09±0.01	0.04±0.01	1.65±0.47	0.18±0.01	0.04±0.01	0.21±0.014
Labrador	132.13±8.5	0.23±0.02	0.04^b^±0.01	0.07±0.01	0.04±0.01	1.41±0.11	0.18±0.01	0.04±0.01	0.23±0.014
Crossbred	109.6±1.14	0.27±0.06	0.04^b^±0.01	0.10±0.02	0.04±0.01	1.30±0.35	0.2±0.01	0.04±0.01	0.21±0.014
Spitz	111.00±9.4	0.26±0.02	0.04^b^±0.01	0.06±0.01	0.04±0.01	1.40±0.10	0.2±0.01	0.04±0.01	0.23±0.014
Beagle	100.00±7.3	0.20±0.03	0.02^b^±0.01	0.04±0.01	0.02±0.01	0.70±0.16	0.2±0.01	0.04±0.01	0.23±0.014
Pug	125.00±10.2	0.23±0.06	0.04^b^±0.01	0.07±0.01	0.04±0.01	0.75±0.26	0.16±0.01	0.04±0.01	0.24±0.014
Dachshund	115.00±12.3	0.20±0.10	0.04^b^±0.01	0.08±0.01	0.04±0.01	1.15±0.25	0.2±0.01	0.04±0.01	0.24±0.014
German Shepherd dog	145.00±5.00	0.25±0.05	0.06^a,b^±0.02	0.08±0.01	0.04±0.01	1.43±0.30	0.18±0.01	0.04±0.01	0.21±0.014
Pomeranian	106.25±3.75	0.28±0.03	0.04^a^±0.01	0.07±0.01	0.04±0.01	1.07±0.31	0.18±0.01	0.04±0.01	0.22±0.014
Golden retriever	140.50±25.5	0.45±0.05	0.08^a^±0.04	0.06±0.02	0.04±0.01	1.70±0.10	0.2±0.01	0.04±0.01	0.21±0.014
Doberman	150.00±18.2	0.30±0.10	0.08^a^±0.02	0.12±0.03	0.04±0.01	1.320±0.1	0.18±0.01	0.04±0.01	0.23±0.014
p-value	0.69	0.42	0.05*	0.1	0.45	0.08	0.43	0.06	0.6
Variation between sex									
Male	120.07±7.88	0.24±0.02	0.04±0.01	0.08±0.01	0.04±0.01	1.34±0.11	0.15±0.01	0.04±0.01	0.22±0.014
Female	118.61±5.13	0.27±0.02	0.04±0.01	0.7±0.01	0.04±0.01	1.28±0.10	0.18±0.01	0.04±0.01	0.21±0.013
p-value	0.87	0.34	0.85	0.06	0.06	0.66	0.31	0.46	0.06
Variation between different age groups									
<6 months	108.75±4.20	0.20±0.04	0.03^b^±0.01	0.06±0.01	0.03±0.01	1.10±0.45	0.2±0.01	0.04±0.01	0.22±0.012
6 months-2.5 years	101.25±5.15	0.23±0.03	0.04^b^±0.01	0.06±0.01	0.04±0.01	1.03±0.20	0.18±0.01	0.04±0.01	0.22±0.012
2.5-4.5 years	134.00±3.23	0.29±0.02	0.04^b^±0.01	0.08±0.01	0.04±0.01	1.02±0.12	0.19±0.01	0.04±0.01	0.22±0.011
4.5-6.5 years	109.67±6.57	0.25±0.04	0.03^b^±0.01	0.07±0.01	0.04±0.01	1.43±0.24	0.2±0.01	0.04±0.01	0.22±0.011
6.5-8.5 years	122.32±7.39	0.27±0.02	0.05^a,b^±0.01	0.06±0.01	0.04±0.01	1.57±0.11	0.21±0.01	0.04±0.01	0.21±0.012
8.5-10.5 years	114.67±5.91	0.22±0.03	0.03^b^±0.01	0.08±0.01	0.04±0.01	1.28±0.20	0.18±0.01	0.04±0.01	0.22±0.012
10.5-12.5 years	122.33±36.6	0.30±0.10	0.07^a^±0.03	0.11±0.01	0.04±0.01	1.20±0.23	0.2±0.01	0.04±0.01	0.21±0.013
12.5-15 years	102.50±2.50	0.25±0.05	0.04^b^±0.01	0.06±0.02	0.04±0.01	1.65±0.15	0.19±0.01	0.04±0.01	0.23±0.013
p-value	0.69	0.66	0.05*	0.1	0.08	0.19	0.54	0.08	0.07

Values are expressed as mean±SE. Values with different superscripts within a column differ significantly (p<0.01)

The variation of heart rate in different breeds of dogs obtained in this investigation may occur due to several factors such as exercise, age, breed, body condition score, motor activity, and sleep [12]. We found slightly lower values in Beagle when compared to that of Hanton and Rabemampianina [13], in the same breed of dogs. The heart rate of GSD obtained in this study was corroborated with the earlier reports of Rezakhani *et al*. [14] but higher than trained GSD reported by Mukherjee *et al*. [9]. The Labrador breeds were exhibiting higher heart rate in this study than the earlier report [8]. The heart rate of Mongrels was within the range as reported by Schneider *et al*. [15]. The variability in the heart rates has been studied earlier among different breeds such as Doberman [16], Spaniels [17], and Beagles [18], both in clinically healthy and diseased model. However, most of the studies considered a single breed rather than comparing different breeds of dogs. In one of our previous investigations [9], we compared the heart rates in trained Labrador, German Shepherd, and Golden Retriever dogs used in police dog squad. In comparison with these data with the present investigation, it was depicted that trained dogs exhibited lower heart rates compared to normal dogs of the same breeds. In a related study, Doxey and Boswood [19] compared GSD, Labrador, Cocker Spaniels, Boxer, Bulldog, and Cavalier King Charles spaniels for heart rate variation and found no significant differences among breeds.

### Heart rhythm

The majority of dogs under study were exhibited normal sinus rhythm (25.40%). The incidence of sinus arrhythmia was 15.87% which was mostly common in older dogs (10.5-12.5 years). Spitz breeds were most susceptible to sinus arrhythmia depicted in this study. Sinus tachycardia was found in 17.46% of dogs under investigation of which Labrador and Spitz were most susceptible and its incidence was maximum in the age group of 2.5-4.5 years and 6.5-8.5 years. The incidence of bradycardia was less (3.17%) with the maximum occurrence in Spitz.

The overall incidence of arrhythmia depicted in the present investigation was slightly lower than the earlier reports of Kumar *et al*. [7] (21.92%) and higher than the reports of Sarita [20] (7.67%). The highest incidences of cardiac arrhythmias were reported in Pomeranian [7,20] but, in our investigation, we found that the incidence was highest in Spitz and Labrador. This could be probably due to less number of observations (2%) in this breed during investigation. The age-wise incidence of cardiac arrhythmias was in accordance with the earlier observation [21] reported a higher incidence of cardiac arrhythmias in pups and older dogs.

### Waves and complexes of ECG

The values of different waves and complexes of ECG in different breeds, age groups, and sex are presented in Table-1. In this investigation, measurements of waves and complexes were made from lead-II as it is directed toward the electrode of lead II and considered as waves of typical depolarization vector [9,10]. We did not get any significant variation (p>0.05) between breeds, age, and sex in the amplitude of P wave, the first positive wave denoting atrial depolarization in lead-I, II, III, and aVF though significant variations (p<0.05) found in P wave duration between different breeds and different age groups. We found no significant differences (p>0.05) between breed and age groups in our previous study [9] on the trained breed of dogs. The values of P wave amplitude were in accordance with the earlier reports of Burman *et al*. [22] but higher than the other reports [8,9,23]. On breed-wise comparison, the values were similar for earlier reports in Beagle [13] and Labrador, but the values for Golden Retriever were lower than the earlier reports [9] and the values were higher for GSD compared to the earlier studies [9,14]. The variation in the P wave amplitude may be in response to heart rate and stress [8]. The sex has no effect on P wave configuration as corroborated by the earlier studies [13]. On contrary, Upeniece [24] showed that P waves in lead-I and aVF were correlated with sex and body weight. The obtained values of PR interval representing the nodal delay and the time required for the electrical impulses to travel from SA node to AV node were supported by earlier works [9,25].

The Q wave is the first negative deflection in lead-II and represents the electrical transmission into intraventricular septum. Earlier report [22] showed the presence of Q wave in lead I, II, III, and aVF and absence in lead aVR and aVL. However, in contrary, Avizeh *et al*. [22] and Bernal *et al*. [26] reported the presence of Q wave in all the leads. We also found Q waves in all the leads without any significant variation in the amplitude between breeds, age, and sex. Bernal *et al*. [26] also reported a non-significant difference between sex and body weight. However, Sato *et al*. [27] reported that altered Q wave amplitude between different breeds may be associated with thoracic peculiarities of different dogs.

In the present study, QRS duration representing ventricular depolarization in the dogs, the increase of which is indicative of ventricular enlargement [25] was similar between breeds, sex, and age groups and in accordance with the earlier reports [9,21].

The R wave represents the depolarization of major ventricular muscles and used to diagnose the left ventricular function [25]. In our current investigation, R wave did not vary significantly between breeds, but non-significant higher values were obtained in Labrador, GSD, Mongrels, and Doberman compared to small breeds. Earlier reports [25] also stated higher R wave amplitude in large breeds of dogs due to increased ventricular surface area and thickened wall in large breeds compared to smaller ones.

The QT intervals obtained in this investigation were in accordance with the earlier reports [8,9]. Normally QT interval is the representative of ventricular systole and it is the summation of ventricular depolarization and repolarization [23]. QT interval varied between wide range and under the influence of catecholamines and vagal activity [23], but, in this investigation, there were no significant differences between different breeds, sex, and age groups.

The repolarization of ventricular myocardium can be represented in T wave and normally it should be less than one-fourth amplitude of R wave [25]. T wave amplitude and duration obtained in this investigation were in accordance with the earlier reports [8,27] but lower than the reports of Gugjoo *et al*. [8]. In this investigation, 44% of dogs exhibited positive T wave and 56% of dogs appeared with inverted T wave. The reasons for altered polarity of T wave were not fully understood [28,29], but some authors suggested that the elevation of diaphragm during respiration could be one of the probable reasons behind the altered polarity of T wave in lead-II [7]. Breed-wise variations in the T wave polarity have also been documented in our previous study [9]. In some dogs, ventricular repolarization occurs in the same direction as depolarization, from inside the ventricles (endocardium) to outside (epicardium). This repolarization pattern generates a negative voltage in the left chest member, compared to the right chest member, forming a deflection of the negative T wave [30].

## Conclusion

The present investigation generates some basic data on canine electrocardiographic patterns in different breeds which expand the existing knowledge to help the clinicians for monitoring cardiac abnormalities in different breeds and different age groups.

## Authors’ Contributions

JM planned the study. SSM, SJ, JM, DB, and KD recorded the data. PKD analyzed the data. JM and PRG drafted and revised the manuscript. All authors read and approved the final manuscript.
